# An accidental liraglutide overdose: case report

**DOI:** 10.3402/ljm.v9.23055

**Published:** 2014-01-20

**Authors:** Rafik R. Elmehdawi, Abdulwahab M. Elbarsha

**Affiliations:** 1 Department of Medicine, Faculty of Medicine, Benghazi University, Benghazi, Libya; 2 Department of Medicine, Benghazi Medical Center, Benghazi, Libya

Liraglutide is a glucagon-like peptide 1 (GLP-1) agonist that is 97% homologous to the endogenous human GLP-1 (1). By mimicking the effects of native GLP-1, it enhances the glucose-dependent secretion of insulin from beta cells of pancreatic islets, suppresses elevated glucagon secretion, and slows down gastric emptying and increases satiety (2). It results in a 0.5–1.5% reduction in HbA1c (3) and can be used as a monotherapy or as an add-on therapy to metformin, sulfonylurea, thiazolinediones, and/or insulin (2). It was approved by the European Medicines Agency in 2009 and by the US Food and Drug Administration in 2010.

We report the case of a 49-year-old woman who presented with an accidental liraglutide overdose. She has type 2 diabetes treated with a prescribed metformin 850 mg t.d.s. liraglutide pen with a starting dose of 0.6 mg per day subcutaneously. Her most recent HbA1c was 8.9%, and her body mass index was 41.6 kg/m^2^. On the first day of liraglutide therapy, she mistakenly injected the whole liraglutide pen by repeating the 0.6 mg dose injection 30 times (total dose of 18 mg).

One hour later she developed nausea and vomiting and her blood glucose at that time was 3.6 mmol/L. She was admitted two and a half hours after the overdose occurred.

She did not complain of abdominal pain but she was severely nauseated and vomiting.

Her blood glucose on admission was 4.49 mmol/L.

In hospital, she was treated with intravenous fluids and intravenous metoclopramide. Her liver function tests and serum amylase levels remained within the normal range. She was aferbril, and her blood pressure was 170/100 mm Hg.

Her nausea improved and vomiting stopped 13 hours after the overdose – she vomited a total of 19 times. Her blood glucose during admission ranged from 4.49 to 12.93 mmol/L (mean 10.1±2.23 mmol/L).

She was discharged 24 hours after admission. For the next five days, the patient felt mildly nauseous and had excessive belching but no abdominal pain; her blood glucose ranged from 6.6 to 9.76 mmol/L without any anti-hyperglycemic therapy. On Day 7, the patient's blood glucose started rising and she restarted her metfromin treatment.

Few cases of liraglutide overdose are reported in the literature (4–6). Severe nausea and vomiting and occasional abdominal pain and diarrhea are the only reported symptoms (4–6). In none of the reported cases did hypoglycemia occur even when the administered dose was massive (72 mg) (5) or the overdose was continued for a long time (seven months) (6). There was no incident of pancreatitis, and the treatment is mainly supportive and aimed at relieving nausea and vomiting.

*Rafik R. Elmehdawi*
Department of Medicine
Faculty of Medicine
Benghazi University
Benghazi, Libya
Department of Medicine
Benghazi Medical Center
Benghazi, Libya *Abdulwahab M. Elbarsha*
Department of Medicine
Faculty of Medicine
Benghazi University
Benghazi, Libya
Department of Medicine
Benghazi Medical Center
Benghazi, Libya
Email: elbarsha@hotmail.com
